# Supplementary material for: Are carbon emissions trading and green financial instruments synergistic? -Comprehensive quantitative research based on content analysis
Source: PLoS One. 2024 Mar 7;19(3):e0298601. doi: 10.1371/journal.pone.0298601 (PMC10919601; doi:10.1371/journal.pone.0298601)
Supplement: S1 Data — (ZIP) [file pone.0298601.s001.zip › Data/地级市Z_F工作报告.zip!/╒π╜¡/╠¿╓▌╩╨/└┤╫╘╣1⁄2╓┌║┼í╛┬φ┐╦╩2╛▌═°í┐_2018.docx]

各位代表：

现在，我代表台州市人民政府，向大会报告政府工作，请予审议，并请市政协委员和其他列席人员提出意见。

一、2017年工作回顾

2017年是本届政府任期的第一年。市政府认真贯彻落实省委省政府和市委的决策部署，坚定不移沿着“八八战略”指引的路子走下去，围绕建设独具魅力的“山海水城、和合圣地、制造之都”目标，拉高标杆、争先进位，勇当排头兵、再创新辉煌，奋力推进“三大历史任务”，圆满完成了市五届人大一次会议确定的目标任务，办成了一些多年想办而没有办成的大事，解决了一些长期想解决而没有解决的难题，取得了新世纪以来最好的成绩单，多项主要指标增速全省领先，生产总值增长8.1%，财政总收入增长12.5%，一般公共预算收入增长11.1%，城乡居民人均可支配收入分别增长8.9%和9.5%。台州正朝着高水平全面建成小康社会胜利前进。

这一年，我们着力深化供给侧结构性改革，产业转型升级迈出新步伐。积极实施先进制造业“十大行动”，制造之都建设扎实推进。规上工业增加值增长11.6%，居全省第一。发力高端制造，天鹰无人机成功下线，航空特色小镇建设进展顺利，机器人产业快速发展。吉利汽车临海产业园提前形成20万辆产能，领克01在路桥基地量产销售，全产业链汽车城建设加快推进。重大产业项目势头喜人，华海制药科技产业园破土动工，华强方特主题公园顺利签约。加大倒逼转型力度，推进“亩均论英雄”。新增制造业单项冠军示范企业3家。滚动实施小微企业三年成长计划，新增小微企业2.6万家，“个转企”3435家，建成小微企业园109个，入驻企业4612家。推进“两化”融合，新增上云企业7800家。深化“三强一制造”建设，启动全国首个智能马桶质量攻坚行动计划，新增国家级产品质量提升示范区3个、“浙江制造”标准17项，新增“浙江制造”认证企业数居全省第一。天台成为全国质量强市示范城市创建城市。温岭列入全省首批军民融合创新示范区创建名单。实施“128”股改上市三年行动计划，出台股改新政和上市新政双10条，新增股份制企业600家、上市企业11家，上市企业总数52家，其中A股上市企业数居全国地级市第四。实施“瞪羚企业”培育计划，首批入选企业33家。加大惠企力度，减免各类税费122亿元。成立台州民营经济学院，突出创二代培训，推动“薪火相传”。推动金融、物流、电商、文化等产业发展，加快建设省级现代服务业集聚示范区，中国模具博览城投用。积极推进旅游强市建设，柔川、南屏景区被评为4A级景区，桃渚景区列入国家级风景名胜区。现代农业“两区”建设成效明显，建成粮食生产功能区7.3万亩、省级现代农业园区2个，全国农业标准化示范区达13个，获省农博会金奖农产品数连续十五年居全省第一，获国字号渔业品牌6个，成为全省唯一省级农机博览会举办地。率先出台绿色食品质量安全责任保险政策，有效构建四级联动的农产品质量安全监管体系。成功创建国家新型职业农民培训示范基地。

这一年，我们着力强化创新驱动，区域发展增添新动力。围绕“1235”战略目标和“五百五十五翻番”工作目标，积极开启“科技新长征”。推进创新大平台建设，国家高新区创建列入考察名单，市中央创新区完成概念性规划编制。深化校地战略合作，浙大台州研究院建设加快推进，与南科大共建台州·深圳创新中心和台州研究院，建成台州北大科技园、耶大基因与细胞治疗研究院。创建省级众创空间3家、省级科技企业孵化器2家。实施高新技术企业和科技型中小微企业双倍增计划，高新技术产业增加值增长16.7%，新认定国家高新技术企业229家、省级科技型企业1183家，新增省级企业研究院21家，入选首批省创新型领军企业2家。新增国家技术创新示范企业1家、省级企业技术中心10家。13家企业入榜全国企业创新能力1000强，居全省第三。发明专利申请量和授权量均增长20%以上。永太科技获全国专利金奖。突出人才强市，出台人才新政30条，政策力度走在全国前列。成功举办台州国际人才合作洽谈大会和中美（台州）国际生物医药发展论坛。建成省级院士专家工作站3家，居全省第二。台州技师学院开工建设，温岭、三门等地技师学院加快建设。

这一年，我们着力项目攻坚，交通等基础设施建设取得重大突破。开展“大抓项目年”活动，固定资产投资增长15.5%，居全省第一。打响项目攻坚擂台赛，举行七批次省、市重大项目集中开工活动，开工项目440个，总投资4270亿元。综合交通建设捷报频传。历经五年奋战，市区内环线建成通车。沿海高速加快建设，乐清湾大桥及接线工程基本建成。杭绍台高铁全线开工。金台铁路、市域铁路S1线、台金高速东延市区连接线、台金高速东延二期、路泽太高架等项目加快建设。高速三门联络线、现代有轨电车、海城路启动建设。头门港区二期码头工程动工。台州机场改扩建项目开工建设，台州机场成为省机场集团一员。甬台温天然气成品油管道投用。建成220千伏电网项目6个。

积极推进“水上台州”建设，22个项目列入省“百项千亿”防洪排涝工程，栅岭汪排涝调蓄工程基本建成，洪家场浦排涝调蓄、东官河综合整治、温岭南排和朱溪水库、方溪水库、盂溪水库、东屏水库等加快建设，永宁江闸强排、大田平原排涝二期等项目开工。大力推进批而未供、供而未用土地处置等专项行动，新增建设用地指标3.4万亩，工业用地供应1.2万亩，数量均居全省第三。

这一年，我们着力统筹城乡发展，美丽台州展现新面貌。坚决推进市区融合发展，规划、财政、土地等管理体制改革取得决定性突破，社保一体化、普通高中一体化、食品检测资源一体化取得实质性成果，三乙医院实现统一管理。建立和完善市区经营性用地有序供地制度，土地利用效益大幅提高。椒江区和台州经济开发区社会管理职责进一步理顺。启动新一轮城市总体规划编制，成为全国城市总体规划编制试点和城市设计试点。中央商务区形象日渐丰满，台州湾产业集聚区东部新区启动区块基本成型，椒江“一江两岸”综合开发强势推进。省级海绵城市试点顺利推进。大力开展新城建“四大破难攻坚”行动，打通市区断头路11条、断头河38条，完成城中村改造拆迁5万多户，开工建设地下综合管廊32公里，12个主要入城口和沿线道路景观提升加快推进。建成中央山体育公园。新增绿道174公里。深化专项治理，“三改一拆”进度居全省前列，小城镇环境综合整治省级样板镇创建数居全省第一。全力推进治水治土治气，提前三年实现“十三五”规划水质目标，黑臭河、垃圾河全面消除，劣V类水剿灭任务圆满完成，永安溪入选首届“全国十条最美家乡河”，土壤污染综合防治先行区建设扎实推进，城市环境空气质量（AQI）优良率全省第一。“四小车”整治卓有成效。公交一卡通顺利实施，新增停车位3.5万个。经过十一年坚持不懈的奋斗，成功创成全国文明城市，市容市貌焕然一新，市民素质大幅提升。天台创成国家卫生县城。仙居成为省示范文明县城。启动公厕、垃圾、物业“三大革命”，智能化改造公厕32座，市生活垃圾焚烧二期项目建成，4179个村开展农村生活垃圾分类处理。美丽乡村建设积极推进，建成精品村140个，培育农家乐特色村（点）290个，评定3A级景区村庄20个，提升美丽乡村公路1077公里。

这一年，我们着力深化改革开放，营商环境更加优化。在全省率先启动“最多跑一次”改革，6959项（主项）事项实现“最多跑一次”，实现率96.1%，其中“零上门”事项1631项。率先开展“多证合一、证照联办”改革，“十一证合一”经验全省推广，“一窗受理、集成服务”改革全省领先，临海国地税联合办税服务获评全国“百优”。获批全国个体工商户登记制度改革试点，新增市场主体11万户。深化国家小微企业金融服务改革创新试验区建设，市金融服务信用信息共享平台二期上线，信保基金实现市域全覆盖，累计承保122亿元，惠及企业6675家，商标专用权质押贷款增长15%，动产质押融资、小微金改国家标准化编制工作启动，我市在国务院召开的全国小微企业金融服务会议上作典型发言。深化民间投资创新综合改革试点，发布PPP“台九条”，PPP模式运作走在全国前列，杭绍台高铁作为全国首条民营资本控股高铁，载入《党的十八大以来大事记》。市县成立行政复议局，成为全国首个全域行政复议体制改革市。组建市城市管理委员会，成立市综合行政执法局（城市管理局），“大城管”格局基本形成。成立市大数据管理中心。国资国企改革稳步推进，台州高速股权集中优化顺利实现。玉环撤县设市。积极推动台州湾区经济发展试验区建设，南、北部湾区管委会挂牌，头门港经济开发区获批省级经济开发区。大麦屿港区集装箱吞吐量突破10万标箱。外贸出口增长18%，居全省第二。全面开展对德合作，中德工业城市联盟第四次全会成功举办，中德（台州）产业合作园启动建设。企业海外并购风起云涌，对外投资增长190%，居全省第一。加强异地商会建设。引进省外资金459亿元。扶贫协作和对口支援工作扎实有效。

这一年，我们着力增进民生福祉，人民群众获得感明显增强。全市财政民生投入440.8亿元，占财政总支出的78.3%，增长13.9%。圆满完成25个为民办实事项目。城镇新增就业人数12.2万人，发放创业担保贷款4.6亿元。高度重视教育事业，教育基建和人均教育投入增速均居全省第一，基础教育质量全省排名向前移位，教育现代化发展指标显著提升。椒江、路桥创成省教育基本现代化区。加快引进优质教育资源，北师大台州实验学校开工建设，台州学院航空工程学院成立，台州职业技术学院中德学院运行。扎实推进“健康台州”建设，深化医药卫生体制改革，积极推进医疗资源“双下沉、两提升”和分级诊疗制度。健康一卡通加快推广。合作共建温医大附属眼视光医院台州分院、台州肿瘤医院，市妇女儿童医院基本建成，市公共卫生中心、市第一人民医院新院区等项目进展顺利。完善全民健身服务体系，创成体育强市。出台和合圣地建设行动纲要，成立和合文化研究院，举办天台山和合文化论坛，新建和合书吧20家，创成中国和合文化之乡。开展台州文化年系列活动，国家公共文化服务体系示范区创建中期督查评估居全国第二，4个作品入选省“五个一工程”优秀作品奖。新建农村文化礼堂273家，累计1206家，总量居全省第一。台州影视产业园开园。举办大陈岛垦荒精神理论研讨会。成立中国冬至文化研究中心。社保扩面提质，户籍人员基本养老保险参保率86.3%、基本医疗保险参保率99.1%。公建民营养老机构35家，新建居家养老服务照料中心419个。获评全国幸福家庭活动示范市，入选“中国最具幸福感城市”。培育“一村一品”扶贫示范村103个，完成异地搬迁1万多人，低收入农户增收15%以上。消除集体经济薄弱村累计达到1339个。深入实施“平安五连创”行动。完善治安防控体系，开展平安台州2017系列行动，强势推进“打霸拔钉”，形成有力震慑。深刻吸取重大火灾事故教训，深入开展“查危除患”大行动，创新推进安全生产和消防整治标准化。推进全科网格、出租房“旅馆式”管理、乡镇“四个平台”建设等工作，社会治理基层基础更加扎实，刑事案件、交通事故死亡人数大幅下降，安全生产控制指标连续十三年下降。启动国家食品安全示范城市创建，放心农贸市场改造顺利完成。扎实推进“七五”普法和公共法律服务体系建设，完成4部地方性法规和规章审查。开展信访积案“歼灭年”活动，信访积案大幅化解，12345市长热线服务水平居全国前列。市区丧葬移风易俗成效明显。深化“一打三整治”专项行动。严厉打击成品油等走私活动。市应急指挥中心和市政府应急平台投用。地质灾害防治三年行动计划全面启动。村（居）委会换届选举率先完成。统计基层基础不断夯实。国防动员水平不断提升，“乡贤回归故里”军民融合座谈会成功召开，全力支持驻台部队全面停止有偿服务，征兵工作量化排名全省第三。外事侨务、对台事务、民族宗教、气象、档案、地方志等工作取得新成绩。妇女儿童、残疾人、老龄、老干部、关心下一代、慈善等工作不断加强。

各位代表！过去一年，市政府始终坚持正确政治方向，牢固树立“四个意识”，落实全面从严治党要求，加快政府职能转变，推进依法行政。认真落实市人大及其常委会决议决定，自觉接受人大依法监督、政协民主监督，认真听取民主党派、人民团体和各界人士意见，办理人大代表议案建议400件、政协委员提案396件，办结满意率100%。坚决纠正“四风”，“三公”经费支出下降37.4%。支持监察体制改革试点工作，强化审计监督，加大惩治腐败力度，政府系统廉政建设全面加强。

各位代表！上述成绩的取得，是省委省政府和市委坚强领导的结果，是市人大及其常委会、市政协有效监督和大力支持的结果，是全市人民励精图治、奋力拼搏的结果。在此，我代表市人民政府，向全市人民和广大建设者，向人大代表、政协委员，各民主党派、工商联、人民团体和社会各界人士，向驻台部队和武警官兵、在台省部属各单位，向关心、支持台州发展的海内外朋友们，表示衷心的感谢！

同时，我们也清醒认识到，面对人民日益增长的美好生活需要，不平衡不充分发展问题仍然突出。产业“低小散”格局还没有根本改变，产业优化升级十分紧迫；新经济短板突出，创新驱动支撑不足，新旧动能转换还需付出更大努力；中心城市辐射带动能力不强，城中村大量存在，城乡统筹发展仍需加强；优质公共服务供给不足，离群众期望还有差距；安全事故易发多发势头还未根本扭转，基层社会治理能力和水平有待提高。我们一定高度重视这些问题，采取更加有力措施认真加以解决。

二、2018年工作任务

今年是贯彻党的十九大精神的开局之年，是决胜全面建成小康社会、实施“十三五”规划承上启下的关键一年，也是改革开放40周年。回首台州波澜壮阔的改革发展历程，我们有过豪迈和辉煌，也有过曲折和坎坷，更有着坚守和执着、奋斗和汗水、成功和喜悦。从1978年到2001年，台州在全国率先推行股份合作制，民营经济高歌猛进，创造了历史的辉煌。从2002年到2007年，台州经济先发优势弱化，遭遇了成长的烦恼。在这一关键节点，时任中共浙江省委书记的习近平同志于2002年12月21日到台州考察，高瞻远瞩地提出“推进台州民营经济发展再创新辉煌”的历史课题。我们开始了为期15年的艰难转型。从2008年到2015年，受金融危机影响，台州经济经受了严峻考验，我们牢记嘱托，稳住阵脚、保持定力，坚守实业、坚定转型，实现了凤凰涅槃。2016年以来，台州经济强势复苏、焕发活力。我们乘势而上，拉高标杆，争先进位，正豪情满怀地续写新的辉煌。

今天的台州，经济质量有了大提升。经济总量突破4000亿元，高端制造布局落子，航空航天、轨道交通和新能源等战略性新兴产业不断壮大。高新技术产业增加值占规上工业45.5%；机电和高新技术产品出口占出口总量62.4%，居全省第一。发展保持中高速，质量迈向中高端。

今天的台州，交通条件有了大变化。一批重大交通项目陆续建成，综合交通体系逐步完善，即将形成市内1小时交通圈，实现联结省内四大都市区1小时交通圈、联结长三角核心城市2小时交通圈，外联内通更加顺畅。

今天的台州，生态环境有了大改善。空气质量稳居全国前列，“台州蓝”成为常态，劣V类水体基本消除，水环境质量明显改观，生态环境质量满意度从2012年全省第十跃居第二，成为“全国环境治理项目推进快，重点区域大气、重点流域水环境质量明显改善”的四个城市之一。

今天的台州，融合发展有了大突破。市区体制基本理顺，一体化步伐加快，基础设施和公共服务共建共享，老百姓切身感受到了融合发展带来的好处。市域发展更加协同，大台州的文化认同、心理认同逐渐增强，“我是台州人”深入人心。

今天的台州，营商环境有了大优化。政府有为和市场有效良性互动，小微金融服务成为全国样板，金融生态和社会信用优良，重商亲商氛围浓厚，政府服务更为高效，纳税人满意度全国地级市排名第一，法治政府建设稳居全国大中城市前列，创业创新“黑土地”更加肥沃。

各位代表！40年来，全市上下锐意改革、开拓创新、奋勇争先，创造了历史性成就，台州大地发生了历史性变化，台州发展迎来了历史性机遇。我们绝不能因为胜利而骄傲，绝不能因为成就而止步，绝不能因为困难而退缩。要继续高举改革大旗，扛起改革担当，乘势而上，真抓实干，创造新的业绩。

各位代表！党的十九大宣告了中国特色社会主义进入新时代。做好今年政府工作，要全面贯彻党的十九大精神，高举习近平新时代中国特色社会主义思想伟大旗帜，认真落实市五届二次党代会决策部署，坚持稳中求进工作总基调，坚持新发展理念，聚焦聚力高质量发展，以供给侧结构性改革为主线，全力打好防范化解重大风险、传统产业优化升级、低收入百姓增收、污染防治攻坚战，奋力推进“三大历史任务”，统筹抓好改革发展稳定各项工作，拉高标杆，争先进位，努力在全省领跑快跑、走在前列，为“两个高水平”建设奠定坚实基础。今年经济社会发展的主要预期目标是，生产总值增长7.5%以上；一般公共预算收入增长、城乡居民收入增长与经济增长基本同步，城乡居民收入比缩小；居民消费价格涨幅3%左右；城镇新增就业6.5万人以上，城镇登记失业率控制在3.5%以内；外贸出口占全国比重9‰以上；研究与试验发展经费支出相当于生产总值2.12%左右；投资结构优化、效益提高；单位生产总值能耗、温室气体控排和污染排放量完成省定目标。

新时代开启了高质量发展新征程。围绕高质量发展，今年我们重点抓好以下七方面工作：

（一）大力推动传统产业优化升级，擦亮高质量发展底色

传统产业是台州最厚实的家底，唯有优化升级才能再创辉煌。大力实施传统产业优化升级三年行动计划，确保一年初见成效、两年基本完成、三年彻底转型。

坚决打破坛坛罐罐。按照“彻底告别‘三合一’、小微企业进园区、民房群租旅馆化、职工住进宿舍里”要求，加快低小散、厂中厂、老旧工业点、老旧市场改造等专项整治。实施新一轮小微企业三年成长计划，加快小微园区建设，实施“313”行动计划，力争三年内建成小微工业园、改造老旧工业区3000万平方米，实现1万家小微企业进园区、30万名职工进宿舍。今年开工建设小微工业园47个。

推动产业质量提升。围绕汽摩配、医药化工、家用电器等十大传统产业，逐一推进优化升级。深化“三强一制造”建设，争创模具、植保与清洗机械等全国知名品牌示范区，推进智能马桶、机电及机械零部件产品国家质检中心建设，争取泵类等产品国家质检中心落户，新增“浙江制造”品牌培育企业40家，通过“浙江制造”品牌认证6家，参与制（修）订国际、国家、行业标准25项，牵头制订“浙江制造”标准8项。加快特色小镇和产业创新综合体建设，推动黄岩电动车、天台交通科技、仙居医疗器械、三门橡胶等产业园建设。推进建筑工业化，提升建筑质量和建设效率。

坚持扶优扶强。实施大企业培育“2211”工程，培育一批航母企业、旗舰企业和瞪羚企业，打造一批细分行业的“单打冠军”和“隐形冠军”。推进企业管理创新和制度创新，积极贯彻落实“凤凰行动”，大力推进“128”股改上市三年行动计划，完成股份制企业改造600家，确保三年任务两年完成。新增报会、上市企业20家以上。积极推进兼并重组。加快建立现代企业制度。重启中国民营经济论坛。传承弘扬优秀企业家精神和工匠精神。研究制定优质企业留驻本土发展的综合措施，降低实体经济成本，让广大企业安心留在台州发展。

（二）积极发展以数字经济为核心的新经济，培育高质量发展动能

主动顺应“中国制造2025”“互联网+”发展新形势、新要求，加快数字与技术对实体经济的提升改造，促进工业化与信息化“两化”深度融合，实现先进制造业与现代服务业双轮驱动。

加快发展信息经济。大力发展云计算、物联网、大数据和人工智能等产业，加快“数字台州”建设，推动“四化”同步协同，争创省级“两化”深度融合示范区。实施“云—网—端”协调发展战略，大力推进网络基础工程、云计算公共服务平台和智能终端感知系统建设。积极筹建新一代信息网络与人工智能产业研究院，启动规划建设未来信息、网络产业小镇，引进和培育一批创新型信息研发与人工智能企业。实施“企业上云”三年行动计划，实现从数量向质量提升。推进电子政务云、智慧云、企业云等平台建设，培育发展云应用服务商，加快发展具有本地产业特色的行业云应用平台，更好满足企业发展需求。

加快发展战略性新兴产业。围绕航空航天、现代轨道交通装备、工业机器人、智慧管网等新兴产业，积极引进布局一批高端制造项目和军民融合项目。加快培育汽车、生物医药等若干个千亿级高端产业集群。建成吉利汽车临海产业园30万辆扩建项目，加快无人机通航产业园、中车配套产业园、吉利发动机和新能源汽车、巨科铝轮毂等项目建设，推动核电一期投运。实施省市县长项目工程，发扬硬着头皮、厚着脸皮、磨破嘴皮、饿着肚皮、踏破脚皮的“五皮”招商精神，着力招大引强，发挥异地商会作用，加强要素保障，提供精准服务。

加快发展现代服务业。深入实施“1568”战略，打造现代金融、休闲旅游、现代物流、电子商务、商贸市场、房地产六大主导产业。大力发展工业设计、信息和软件服务业、科技服务等生产性服务业，建设模塑工业设计基地三期。发展平台经济、分享经济、体验经济和创意经济，形成与台州制造相融合的现代服务业体系。编制全域旅游发展规划，台州府城文化旅游区创成5A级景区，积极推动长屿硐天创建5A级景区、神仙居创建国家级旅游度假区，加快石塘半岛、三门蛇蟠景区建设，支持天台、仙居争创省级全域旅游示范区，注重发展都市旅游和智慧旅游。打造百家商贸流通企业和十大展会，创建一批城市智慧商圈和现代商贸特色镇，抓好电商集聚和跨境电商集群发展，推进消费升级，深入创建放心消费城市，推动生活性服务业向精细化和高品质转变。发展健康养老、体育休闲等产业。

（三）大踏步走好“科技新长征”，强化高质量发展战略支撑

创新是引领发展的第一动力，是建设现代化经济体系的战略支撑。深入实施创新驱动战略，扎实推进“科技新长征”，争创国家创新型试点城市。

建设创新平台。全力以赴创成国家高新区，加快建设台州科技城，跻身浙东南国家自主创新示范区，构建高能级创新平台和高水平国际产业合作平台。加快市中央创新区建设，力争台州湾创新广场等项目开工，建设人才社区、梦想加速区等，打造科创核心区。加强与大院名校共建创新载体，建成浙大工程师学院台州分院、南科大台州研究院、浙工大台州研究院，推进北航研究院建设。

壮大创新主体。深入实施高新技术企业和科技型中小微企业双倍增计划，推进“百企示范、千企试点、万企行动”，培育一批具有创新能力的排头兵企业，新认定国家高新技术企业126家、省级科技型中小企业502家，新增企业研发机构108家。优化布局新型人才研发机构，建成台州医学健康（新药临床）研究院。继续建设一批众创空间、科技企业孵化器。

优化创新生态。深化人才新政，打造人才生态最优市。引育一批带技术带项目的领军型创业创新人才团队，加强企业家、科技人才、高技能工匠等人才队伍建设，加大跨境人才和项目引进力度。完善高端人才中介机构和人才服务联盟运行机制，加快人力资源服务产业园建设。发挥科技大市场、国际技术转移中心等作用，扩大国际科技交流和军民科技合作。大力引进培育科技服务机构，促进科技成果转化。加强知识产权强市建设，加大专利和商业秘密保护力度。

（四）深入实施乡村振兴战略，开启高质量发展农业农村新局面

坚持农业农村优先发展，按照产业兴旺、生态宜居、乡风文明、治理有效、生活富裕总要求，全力打造农业强、农村美、农民富的新乡村。

推动农业全面升级。深化农业供给侧结构性改革，走质量兴农之路，加快农业现代化，让农业成为有奔头的产业。落实粮食安全县（市、区）长责任制，稳定粮食生产，确保粮食总产量62万吨。推动农业由增产导向转向提质导向，加快省级现代农业园区、特色农业强镇建设，打造一批主题鲜明的田园综合体。大力发展观光农业、体验农业、创意农业等新业态，推进一二三产业融合发展。全面实施农作制度改革，加强农业高新技术研究推广，加快现代种业发展。启动实施与省农科院的第二批院地合作项目。开展农产品区域公用品牌建设，积极创建国家农产品质量安全放心市。扎实推进海洋与渔业“两区一县”创建，发展绿色生态渔业，推进渔场修复振兴，创建国家渔船管理综合改革试验区。

加快农村全面进步。走城乡融合发展之路，加快形成工农互促、城乡互补、全面融合、共同繁荣的新型工农城乡关系，让农村成为安居乐业的美丽家园。走乡村绿色发展之路，扎实推进“千村景区化”工程，打造A级景区村庄100个，培育乡村振兴示范村20个、美丽乡村精品村20个和示范乡镇10个，启动建设省级美丽宜居示范村4个，加强古村落保护。持续开展“森林乡村”创建。推动农村基础设施建设提档升级，加快“四好”农村路建设，提升改造公路300公里，建设美丽公路示范路800公里，创建美丽公路示范乡镇9个。实施全域土地整治“341”工程。抓好农业面源污染治理。实施农村生活垃圾分类处理三年行动，打造农村生活垃圾分类处理示范村100个。走乡村文化兴盛之路，提升乡镇综合文化站服务效能，在1500人以上建制村新建农村文化礼堂150家，弘扬和合文化，提高乡村社会文明程度。走乡村善治之路，坚持自治、法治、德治相结合，创新乡村治理体系，加强农村基层基础工作。

促进农民全面发展。走共同富裕之路，加强支农惠农，扶持农民，提高农民，让农民成为有吸引力的职业。优化农村金融服务，推进农业保险提标扩面。深化农村集体产权制度改革，确保完成确权成果综合验收。拓展“三位一体”农合联改革成果。深化万名乡贤帮千村活动和强基惠民“村村帮”工程，壮大农村集体经济，全面消除集体经济薄弱村。拓宽农民增收渠道，大力发展乡村旅游、电子商务等美丽经济，搭建农产品网络营销平台，积极发展民宿型农家乐特色村，让美丽经济成为农民致富的新增长点。加快全国新型职业农民培育示范基地建设，积极培育新型农业经营主体。建立乡村振兴学院，培养乡村振兴实用人才3万人。走中国特色减贫之路，坚持精准帮扶，强化就业创业扶持，实施低收入农户奔全面小康计划，确保低收入农户人均可支配收入增长10%以上。

（五）统筹推进港产城湾一体化发展，构建高质量发展系统平台

认真落实省大湾区大通道大花园大都市区行动纲要，发挥台州湾区资源丰富的优势，坚持规划引领、陆海统筹、三湾联动、融合发展，高水平建设台州湾区经济发展试验区，打造以“港口为龙头、产业为支撑、城市为主体、湾区为依托”的现代化湾区都市。

构建以头门港区为核心的港口群。整合港口资源，完善集疏运体系，完成头门港区一期码头滚装功能改造，加快二期码头工程，启动疏港公路复线工程，实现水陆域开放，积极申报台州综合保税区。支持大麦屿港区与宁波舟山港深度合作。规划建设相关港区油气登陆配套码头、集装箱码头和进港航道等。落实习近平总书记建设“小康的大陈、现代化的大陈”指示，加快大陈航运集聚区建设，开通金清至大陈航线。加快建设立体交通网。建成沿海高速和台金高速东延二期工程。加快建设杭绍台高铁、金台铁路、杭绍台高速、台金高速东延市区连接线、杭温高铁等项目。开工建设高速温岭联络线、甬台温高速城南互通等项目。加快杭绍台高铁温岭至玉环段项目前期，争取年内开工。推动沿海高铁、金台城际铁路等项目前期。加快台州机场改扩建，推进机场高架等周边路网建设，打造机场综合交通枢纽。开展海港铁联运前期工作。

构建以台州湾产业集聚区为主平台的临港产业集群。以台州湾产业集聚区为核心，构建联动发展、联动招商机制。完善三湾六板块产业布局，形成集聚区东部组团、临海东部、温岭东部、台州南部湾区、台州北部湾区、天台苍山六大产业平台。大力发展海洋经济，科学布局建设若干临港产业园，形成海洋战略性新兴产业集聚集群发展平台，打造蓝色发展新引擎。以“高端、智能”为导向，积极发展大交通、大健康、大装备产业，重点发展汽车及零部件、医药制造、核关联产业、轨道交通、通用航空、缝制设备等，打造若干个世界级产业集群和国家级产业基地。

构建三区三市协同、三县联动发展的城市群。抓好城市总体规划编制和城市设计两个国家试点，按照全市域统筹要求，力争完成新一轮城市总体规划报批。基本建成空间规划信息平台，推进“多规合一”。启动铁路中心站新区开发，加速椒江“一江两岸”综合开发，推进中央商务区、商贸核心区、永宁江风貌带、飞龙湖生态区、月湖绿岛等区块建设。开工建设新国际会展中心、市委党校迁建等项目，新行政服务中心投用。推进城市有机更新，实施城中村改造159个，其中市区70个，开展内环线两侧区块改造提升，加快开发区东扩区块和城市旧城改造，做好历史文化街区、历史建筑保护及历史风貌修复，推进街巷美化提升。研究市域快速路网布局，优化城市道路建设，推进市域铁路S1线、现代有轨电车、路泽太高架、现代大道、海城路等建设，加快内环线连接线建设，改善慢行系统，发展市域公交，推进绿道网建设。加快建设海绵城市和地下综合管廊。加快市区天然气高压输配管网建设，实现市区管输天然气全面通气。加快实施水利“五大百亿工程”，建成洪家场浦排涝调蓄、东官河综合整治、大田平原排涝一期等工程，推进朱溪水库、方溪水库、台州三期供水等工程，开工建设台州南部湾区引水工程和灵江建闸引水工程。实施城市精细化管理，加快智慧城市建设，推进公厕、垃圾、物业“三大革命”，新（改）建一批城市公厕，打造城市休憩驿站；开展垃圾处理设施建设三年攻坚行动和城市生活垃圾分类处理；创新物业管理体制，加强小区业委会建设。推进“三改一拆”，创成1个无违建县（市、区）、4个基本无违建县（市、区）。构建常态化管理机制，巩固深化全国文明城市创建成果，提升市民素养。加快市区西部山区保护性开发。推进副中心城市和美丽县城建设，加快小城市特色化发展，做大做强中心镇，加强小城镇环境综合整治。

坚持三湾联动、生态优先，打造宜居宜业宜游的现代化幸福湾区。建立健全协调机制，加强三湾各类规划衔接。推进交通基础设施共建共享。加快产业联动，推进南、北部湾区发展。大力发展滨海旅游业，以海岸线和海岛链为轴线，串联融合台州湾区各景点，打造台州海上大花园。加强重点生态功能区、生态敏感区保护，着力构筑湾区发展生态屏障。坚持海陆同防同治，推进海岸线整治修复工程，重要湾口、岛屿、岸段实施生态化、景观化、整体化治理，加强入海排污口规范整治和近海海域环境整治，创建湾区经济生态发展示范市。全面推进“811”美丽台州建设行动，积极创建生态文明建设示范区。认真抓好中央环保督察问题整改落实。推进“五水共治”，严格落实河长制、湾（滩）长制，积极推进“污水零直排区”创建工作，加强精细化截污，实施污水处理设施建设三年攻坚行动，城镇污水处理厂基本实现准Ⅳ类排放，开展中水回用，完成国家水十条考核任务。以美丽河湖市创建为抓手，开展河湖疏浚清淤、生态修复、通路造景、科学调蓄、生态补水，建设秀水美河。加快国家土壤污染综合防治先行区建设，完成土壤污染详查，推进重点区块土壤污染风险管控和治理修复。加快危废设施建设。推进“五气共治”，确保城市空气质量稳居全国前列。实施大规模绿化行动，深化省级珍贵彩色森林示范市建设。实施最严格的水资源管理，深化长潭水库全国良好湖泊环境保护试点，加强饮用水源保护。加大生态补偿力度。创建国家水土保持生态文明示范市。支持仙居国家公园试点。支持天台争创国家生态文明建设示范县。

（六）持续深化改革扩大开放，激发高质量发展活力

坚持以“最多跑一次”改革为牵引撬动经济社会各领域改革，以“一带一路”为统领全面扩大开放，再创体制机制新优势。

深化“最多跑一次”改革。以钉钉子精神，将“最多跑一次”改革进行到底，实现群众和企业办事“最多跑一次、最近跑一次、最好不要跑”，争当全省“最多跑一次”改革排头兵。推进“最多跑一次”改革标准化、规范化建设，推广“一窗受理、集成服务”，年底前100%民生事项和企业事项实现“一次办结”，50%以上民生事项实现“一证通办”。实施“受办”分离改革，探索智慧化受理和审批，推进政务服务“全网办、一证办、移动办、自助办”。完善市、县、镇、村四级政府服务平台，推进乡镇（街道）自助终端全覆盖。深化投资项目“最多跑一次”改革，努力实现项目开工前“最多100天”、竣工验收前“最多跑一次”。实施个体工商户登记制度改革试点，推进“证照分离”，按照应纳尽纳原则推进“多证合一”，推动线上线下“证照联办”，激发大众创业创新活力。

深化小微金改等国家级改革试点。加快小微企业金融服务改革创新试验区建设，全面推进专利权质押，加快推进动产质押融资及小微金融服务标准化试点，探索科技金融合作新模式，构建新型小微企业联合辅导体系，优化金融生态环境，更好服务实体经济，打造金融安全示范区。加快社会信用体系示范城市创建，实施“1135”计划，建立守信联合奖励、失信联合惩戒工作机制。深化民间投资创新综合改革，巩固和扩大PPP“两个常态”成果，激发民间投资活力。深入推进循环经济示范城市、新型城镇化等试点。

统筹推进其他领域改革。深化市区财政、土地体制改革，推动市区水务一体化实质性突破，实现食品检测资源一体化。实施“标准地”制度，推行“区域能评、环评+区块能耗、环境标准”机制，促进要素配置市场化改革，加快形成“亩均论英雄”机制。推进“飞地经济”发展。全面推开林权、海域使用权不动产登记。深化国资国企改革，推进经营性事业单位改革。加快国地税征管体制改革。推进户籍制度改革，加快农业转移人口市民化。

构建全面开放新格局。坚持开放强市战略，深度参与“一带一路”建设，积极对接上海自贸区，北融宁波、南联温州、西承金义、东接海上丝路，着力构建浙江“一带一路”倡议枢纽的重要节点。实施对外开放“515”行动计划，重点支持500家外贸企业做大做强，加快省级跨境电商园区等10个对外开放平台建设，推动白俄罗斯台州汽配园、埃塞俄比亚台州鞋帽园等5个境外经贸合作园区建设。全面深化对德合作，加快中德（台州）产业合作园建设。实施外贸优进优出专项行动，扩大高端装备等进口，优化外贸出口结构，发展加工贸易和服务贸易，支持企业设立海外仓和体验店，新增有外贸出口实绩企业500家以上。支持玉环创建国家级对台经贸合作区。深化山海协作，做好扶贫协作和对口合作、支援帮扶工作。

（七）加快补齐民生短板，共享高质量发展成果

全面落实富民惠民安民行动计划，扎实办好民生实事、关键小事，使人民获得感、幸福感、安全感更加充实、更有保障、更可持续。

优先发展教育事业。美好生活从美好教育开始。多渠道加大教育投入，推进教育现代化市创建，临海、温岭、玉环、仙居创成省教育基本现代化县（市、区）。落实立德树人根本任务，推进素质教育，全面提高基础教育质量，加快提升学生体质健康水平。实施“学前教育发展第三个三年行动计划” ，大力提高公办幼儿园比例，新（改、扩）建二级以上幼儿园36所。推动义务教育城乡一体化发展。促进本地高校内涵式发展，推进台州学院向应用型大学转型，加快台州职业技术学院、台州电大等学校二期工程建设。积极引进优质高端教育资源，加快航空工程学院和长鹰无人机学院建设，探索建设台州学院、台州科技职业学院与北航台州通航产业融合发展的教育共同体。积极构建现代职业教育体系，加快市、县两级职教园区建设。发展继续教育和网络教育。

落实“健康台州”战略。制定实施医疗卫生“补短板”行动计划，深化医药卫生体制改革，促进医保、医疗、医药、医院、医生、中医等“六医”统筹，深入推进医疗资源“双下沉、两提升”。积极探索医联体建设，做好路桥省级医联体试点工作。加快智慧医疗建设，引进一批优质医疗资源，实现健康一卡通全覆盖。推进基层全科医生队伍建设。大力发展中医药事业。推进台州医院新院区、台州浙东医院等项目建设，开展市中医院迁建、台州护士学校等项目前期工作。加强公共卫生事业发展，开展卫生乡镇创建。实施食品安全十大攻坚行动计划，积极创建国家食品安全示范城市。实施全民健身“151”工程，大力发展群众体育、老年体育，提升竞技体育，积极备战省运会。

推动文化繁荣。创成国家公共文化服务体系示范区。开展2018台州文化年系列活动，实施文化惠民、文艺精品创作、文艺人才培育等工程。加强和合文化研究，深入开展和合文化标志地、传播地、示范地建设，推进和合书院、和合书吧等一批主题公共文化设施建设，谋划建设全国和合文化研究中心。推进城市文化公园、社区文化家园、企业文化俱乐部建设，健全文化设施网络。构建“1+6”文化产业体系，大力发展文化创意设计、影视文化产业，推进心海文化生态园等建设。加强下汤文化遗址保护开发。做好台州乱弹等非物质文化遗产传承保护。加快推进大剧院建设，加快美术馆、非遗馆、非国有博物馆群和县（市、区）档案馆新馆建设。推动全民阅读，加强科学普及。

抓好就业和社会保障。落实就业优先战略和积极就业政策，实现更高质量和更充分的就业。提升公共就业服务水平，鼓励创业带动就业，做好高校毕业生、农村转移劳动力、残疾人等重点群体创业就业，动态消除“零就业家庭”。深入开展“台州无欠薪”行动，推进和谐劳动关系创建。全面实施全民参保计划，积极推进全市社保一体化。深化医保制度改革，完善跨省医保异地联网结算。加快建立多主体供给、多渠道保障、租购并举的住房制度。实施公共安全综合保险。推进工资制度改革。加快发展老龄事业和产业，完善养老服务体系，推广养老机构“公建民营”，促进医养融合发展，开工建设市社会福利院。巩固丧葬移风易俗成果，加快台州殡仪馆改扩建。开展全国第四次经济普查。做好双拥创建和优抚安置工作。加强民族宗教、外事侨务工作。进一步做好慈善、残疾人工作。

创建平安台州。争取“平安六连创”，勇夺平安银鼎。坚持强势治安理念，深化平安台州系列行动，大力推进“云上公安、智能防控”战略，深化全科网格，健全治安防控和公共安全体系。树立安全发展理念，完善安全生产责任制，推进“五战联合”除火患全民大行动，构建覆盖城乡的火灾消防防控体系，深化预防道路交通事故全民大行动，持续开展渔业船舶、建筑施工、城镇燃气等重点领域专项整治，坚决遏制重特大事故发生，打赢消防安全翻身仗，确保安全生产控制指标持续下降。加强法律援助和司法救助等公共法律服务体系建设。推进信访法治化建设，完善社会矛盾源头预防和调处化解综合机制。加强基层政权建设，深入推进基层治理体系“四个平台”建设。完善应急体系，提升防灾减灾救灾能力，实施标准化、智慧化、社会化防汛，全面消除地质灾害隐患点。大力支持驻台部队建设，加强国防动员工作，推进民兵调整改革，进一步做好双拥创建和优抚安置工作。

各位代表！市政府高度重视为民办实事项目，经过公开征集、网上评选和反复征求意见，提出了包括敬老爱老、基本医疗、基础教育、公共文化、交通出行、食品安全、旅游惠民、消防安全、城乡环境、全民健身、应急救护、公共安全、便民服务等候选项目，提请会议票决。

三、加强政府自身建设

深入学习贯彻党的十九大精神，以“两强三提高”为导向，加快建设人民满意的政府，为高质量发展提供坚强保障。

建设学习型政府，保持政治坚定。切实运用习近平新时代中国特色社会主义思想武装头脑，在学懂弄通做实上下功夫。认真开展“不忘初心、牢记使命”主题教育，牢固树立“四个意识”，自觉维护以习近平同志为核心的党中央权威和集中统一领导。全面增强学习本领、政治领导本领、改革创新本领、科学发展本领、依法执政本领、群众工作本领、狠抓落实本领和驾驭风险本领，推进政府治理体系和治理能力现代化。

建设服务型政府，牢记为民宗旨。坚持以人民为中心，把提高政府效率作为加强政府自身建设的关键环节，推动各级政府精简行政权力、优化办事流程、实施互联网+政务，高质量履行公共服务、市场监管、社会管理、环境保护等职责，优化营商环境，营造亲清新型政商关系。

建设法治型政府，坚持依法行政。自觉接受市人大法律监督、市政协民主监督，认真办理人大代表议案建议、政协委员提案。健全依法决策机制，积极发挥法律顾问等专家团队决策咨询作用。完善行政执法体制，落实行政执法责任制。加强审计监督。全面深化政务公开，主动接受社会监督、舆论监督。推动各级政府诚信施政，提高政府公信力。

建设务实型政府，强化实干担当。大力弘扬红船精神和大陈岛垦荒精神，始终保持昂扬的精神状态，强化实绩导向，只争朝夕、奋勇争先。大兴调查研究之风，提升谋事、干事、成事能力。落实重点工作清单制度，强化督查考核，铆紧各方责任，健全激励机制和容错纠错机制，给干事者鼓劲，为担当者撑腰，以实干推动发展，以实干赢得未来。

建设清廉型政府，全面从严治政。严格落实全面从严治党主体责任，深化标本兼治，深入推进廉政建设。高标准落实中央八项规定及实施细则精神和省委、市委实施办法，坚持不懈纠正“四风”，认真整治形式主义、官僚主义，坚决治理庸政、懒政、怠政。聚焦重点领域，严肃查处侵害群众利益的不正之风和腐败问题，打造风清气正的政务生态。

各位代表！幸福是奋斗出来的！让我们更加紧密地团结在以习近平同志为核心的党中央周围，在中共台州市委的坚强领导下，始终保持一往无前的奋斗姿态，不驰于空想、不骛于虚声，勇于担当、真抓实干，全力推动高质量发展，为加快建设独具魅力的“山海水城、和合圣地、制造之都”，奋力谱写“两个高水平”台州篇章而努力奋斗！
